# Supplementary material for: Resolving the full spectrum of human genome variation using Linked-Reads
Source: Genome Res. 2019 Apr;29(4):635–45. doi: 10.1101/gr.234443.118 (PMC6442396; doi:10.1101/gr.234443.118)
Supplement: Supplemental Material [file supp_gr.234443.118_Supplemental_longranger-2.2.zip › longranger-2.2.tar.bz2!/lr2.2-public/lib/stan/cmdstan/stan/doc/api/html/statement__2__grammar__def_8hpp_source.html]

Stan: src/stan/gm/grammars/statement\_2\_grammar\_def.hpp Source File


|  |  |
| --- | --- |
|  | Stan  2.5.0  probability, sampling & optimization |


- Main Page
- Related Pages
- Namespaces
- Classes
- Files

- File List
- File Members

All Classes Namespaces Files Functions Variables Typedefs Enumerations Enumerator Friends Macros Pages

- src
- stan
- gm
- grammars

statement\_2\_grammar\_def.hpp

Go to the documentation of this file.

1 #ifndef STAN\_\_GM\_\_PARSER\_\_STATEMENT\_2\_GRAMMAR\_DEF\_\_HPP

2 #define STAN\_\_GM\_\_PARSER\_\_STATEMENT\_2\_GRAMMAR\_DEF\_\_HPP

3

4 #include <cstddef>

5 #include <iomanip>

6 #include <iostream>

7 #include <istream>

8 #include <map>

9 #include <set>

10 #include <sstream>

11 #include <string>

12 #include <utility>

13 #include <vector>

14 #include <stdexcept>

15

16 #include <boost/spirit/include/qi.hpp>

17 // FIXME: get rid of unused include

18 #include <boost/spirit/include/phoenix\_core.hpp>

19 #include <boost/spirit/include/phoenix\_function.hpp>

20 #include <boost/spirit/include/phoenix\_fusion.hpp>

21 #include <boost/spirit/include/phoenix\_object.hpp>

22 #include <boost/spirit/include/phoenix\_operator.hpp>

23 #include <boost/spirit/include/phoenix\_stl.hpp>

24

25 #include <boost/lexical\_cast.hpp>

26 #include <boost/fusion/include/adapt\_struct.hpp>

27 #include <boost/fusion/include/std\_pair.hpp>

28 #include <boost/config/warning\_disable.hpp>

29 #include <boost/spirit/include/qi.hpp>

30 #include <boost/spirit/include/qi\_numeric.hpp>

31 #include <boost/spirit/include/classic\_position\_iterator.hpp>

32 #include <boost/spirit/include/phoenix\_core.hpp>

33 #include <boost/spirit/include/phoenix\_function.hpp>

34 #include <boost/spirit/include/phoenix\_fusion.hpp>

35 #include <boost/spirit/include/phoenix\_object.hpp>

36 #include <boost/spirit/include/phoenix\_operator.hpp>

37 #include <boost/spirit/include/phoenix\_stl.hpp>

38 #include <boost/spirit/include/support\_multi\_pass.hpp>

39 #include <boost/tuple/tuple.hpp>

40 #include <boost/variant/apply\_visitor.hpp>

41 #include <boost/variant/recursive\_variant.hpp>

42

43 #include <stan/gm/ast.hpp>

44 #include <stan/gm/grammars/whitespace\_grammar.hpp>

45 #include <stan/gm/grammars/expression\_grammar.hpp>

46 #include <stan/gm/grammars/statement\_grammar.hpp>

47 #include <stan/gm/grammars/statement\_2\_grammar.hpp>

48 #include <stan/gm/grammars/common\_adaptors\_def.hpp>

49

50

51 namespace stan {

52

53  namespace gm {

54

55  struct add\_conditional\_condition {

56  template <typename T1, typename T2, typename T3, typename T4>

57  struct result { typedef void type; };

58  void operator()(conditional\_statement& cs,

59  const expression& e,

60  bool& pass,

61  std::stringstream& error\_msgs) const {

62  if (!e.expression\_type().is\_primitive()) {

63  error\_msgs << "conditions in if-else statement must be primitive int or real;"

64  << " found type=" << e.expression\_type() << std::endl;

65  pass = false;

66  return;

67  }

68  cs.conditions\_.push\_back(e);

69  pass = true;

70  return;

71  }

72  };

73  boost::phoenix::function<add\_conditional\_condition> add\_conditional\_condition\_f;

74

75  struct add\_conditional\_body {

76  template <typename T1, typename T2>

77  struct result { typedef void type; };

78  void operator()(conditional\_statement& cs,

79  const statement& s) const {

80  cs.bodies\_.push\_back(s);

81  }

82  };

83  boost::phoenix::function<add\_conditional\_body> add\_conditional\_body\_f;

84

85

86

87  template <typename Iterator>

88  statement\_2\_grammar<Iterator>::statement\_2\_grammar(variable\_map& var\_map,

89  std::stringstream& error\_msgs,

90  statement\_grammar<Iterator>& sg)

91  : statement\_2\_grammar::base\_type(statement\_2\_r),

92  var\_map\_(var\_map),

93  error\_msgs\_(error\_msgs),

94  expression\_g(var\_map,error\_msgs),

95  statement\_g(sg)

96  {

97  using boost::spirit::qi::\_1;

98  using boost::spirit::qi::char\_;

99  using boost::spirit::qi::lit;

100  using boost::spirit::qi::\_pass;

101  using boost::spirit::qi::\_val;

102

103  using boost::spirit::qi::labels::\_r1;

104  using boost::spirit::qi::labels::\_r2;

105  using boost::spirit::qi::labels::\_r3;

106

107  // \_r1 true if sample\_r allowed (inherited)

108  // \_r2 source of variables allowed for assignments

109  // set to true if sample\_r are allowed

110  statement\_2\_r.name("statement");

111  statement\_2\_r

112  %= conditional\_statement\_r(\_r1,\_r2,\_r3)

113  ;

114

115

116  conditional\_statement\_r.name("if-else statement");

117  conditional\_statement\_r

118  = lit("if")

119  > lit('(')

120  > expression\_g(\_r2)

121  [add\_conditional\_condition\_f(\_val,\_1,\_pass,

122  boost::phoenix::ref(error\_msgs\_))]

123  > lit(')')

124  > statement\_g(\_r1,\_r2,\_r3)

125  [add\_conditional\_body\_f(\_val,\_1)]

126  > \* (( lit("else")

127  >> lit("if") )

128  > lit('(')

129  > expression\_g(\_r2)

130  [add\_conditional\_condition\_f(\_val,\_1,\_pass,

131  boost::phoenix::ref(error\_msgs\_))]

132  > lit(')')

133  > statement\_g(\_r1,\_r2,\_r3)

134  [add\_conditional\_body\_f(\_val,\_1)]

135  )

136  > - (lit("else")

137  > statement\_g(\_r1,\_r2,\_r3)

138  [add\_conditional\_body\_f(\_val,\_1)]

139  )

140  ;

141

142  }

143

144  }

145 }

146 #endif

stan::gm::add\_conditional\_condition

**Definition:** statement\_2\_grammar\_def.hpp:55

stan::gm::statement

**Definition:** ast.hpp:634

stan::gm::add\_conditional\_body::result

**Definition:** statement\_2\_grammar\_def.hpp:77

stan::gm::add\_conditional\_condition::operator()

void operator()(conditional\_statement &cs, const expression &e, bool &pass, std::stringstream &error\_msgs) const

**Definition:** statement\_2\_grammar\_def.hpp:58

stan::gm::statement\_2\_grammar::statement\_2\_grammar

statement\_2\_grammar(variable\_map &var\_map, std::stringstream &error\_msgs, statement\_grammar< Iterator > &sg)

**Definition:** statement\_2\_grammar\_def.hpp:88

stan::gm::statement\_2\_grammar::statement\_2\_r

boost::spirit::qi::rule< Iterator, statement(bool, var\_origin, bool), whitespace\_grammar< Iterator > > statement\_2\_r

**Definition:** statement\_2\_grammar.hpp:53

stan::gm::conditional\_statement::conditions\_

std::vector< expression > conditions\_

**Definition:** ast.hpp:730

stan::gm::statement\_2\_grammar::error\_msgs\_

std::stringstream & error\_msgs\_

**Definition:** statement\_2\_grammar.hpp:36

stan::gm::add\_conditional\_condition\_f

boost::phoenix::function< add\_conditional\_condition > add\_conditional\_condition\_f

**Definition:** statement\_2\_grammar\_def.hpp:73

stan::gm::statement\_grammar

**Definition:** statement\_2\_grammar.hpp:19

stan::gm::statement\_2\_grammar

**Definition:** statement\_2\_grammar.hpp:22

stan::gm::conditional\_statement

**Definition:** ast.hpp:729

expression\_grammar.hpp

stan::gm::statement\_2\_grammar::conditional\_statement\_r

boost::spirit::qi::rule< Iterator, conditional\_statement(bool, var\_origin, bool), whitespace\_grammar< Iterator > > conditional\_statement\_r

**Definition:** statement\_2\_grammar.hpp:47

statement\_grammar.hpp

stan::gm::add\_conditional\_body::result::type

void type

**Definition:** statement\_2\_grammar\_def.hpp:77

stan::gm::add\_conditional\_body\_f

boost::phoenix::function< add\_conditional\_body > add\_conditional\_body\_f

**Definition:** statement\_2\_grammar\_def.hpp:83

statement\_2\_grammar.hpp

stan::gm::add\_conditional\_body

**Definition:** statement\_2\_grammar\_def.hpp:75

stan::gm::conditional\_statement::bodies\_

std::vector< statement > bodies\_

**Definition:** ast.hpp:731

stan::gm::variable\_map

**Definition:** ast.hpp:439

stan::gm::statement\_2\_grammar::expression\_g

expression\_grammar< Iterator > expression\_g

**Definition:** statement\_2\_grammar.hpp:39

stan::gm::add\_conditional\_body::operator()

void operator()(conditional\_statement &cs, const statement &s) const

**Definition:** statement\_2\_grammar\_def.hpp:78

stan::gm::statement\_2\_grammar::statement\_g

statement\_grammar< Iterator > & statement\_g

**Definition:** statement\_2\_grammar.hpp:40

stan::gm::expression

**Definition:** ast.hpp:216

stan::gm::add\_conditional\_condition::result

**Definition:** statement\_2\_grammar\_def.hpp:57

ast.hpp

stan::math::e

double e()

Return the base of the natural logarithm.

**Definition:** constants.hpp:86

whitespace\_grammar.hpp

common\_adaptors\_def.hpp

stan::gm::add\_conditional\_condition::result::type

void type

**Definition:** statement\_2\_grammar\_def.hpp:57

stan::gm::expression::expression\_type

expr\_type expression\_type() const

**Definition:** ast\_def.cpp:570

stan::gm::expr\_type::is\_primitive

bool is\_primitive() const

**Definition:** ast\_def.cpp:93


---

[ Stan Home Page ]

*© 2011–2014,
Stan Development Team.*
